# Automated, Radiation-Free Detection of Cochlear Implant Electrode Array Migration Using Routine Impedance Telemetry

**DOI:** 10.1007/s10916-026-02468-0

**Published:** 2026-11-04

**Authors:** Stephan Schraivogel, Tobias Rader, Tobias Weissgerber, Marco Caversaccio, Wilhelm Wimmer

**Affiliations:** 1 Department of Otorhinolaryngology, Head and Neck Surgery, Inselspital, University of Bern, Bern, Switzerland; 2 ARTORG Center, University of Bern, Bern, Switzerland; 3 Department of Otorhinolaryngology, TUM University Hospital, Technical University of Munich, Munich, Germany; 4 Department of Otorhinolaryngology, LMU University Hospital, LMU Medizin, Ludwig-Maximilians-Universität München, Munich, Germany; 5 Audiological Acoustics, Department of Otorhinolaryngology, University Hospital, Goethe University Frankfurt, Frankfurt am Main, Germany

**Keywords:** Neural prosthetics, non-invasive follow-up, telehealth, objective measures, decision support

## Abstract

Electrode array migration is a rare condition after cochlear implantation that can impair auditory outcomes but often remains undetected during routine follow-up. We evaluated the clinical utility of an electrical impedance-based, machine learning method for detecting postoperative electrode array migration using routinely acquired telemetry data. In this retrospective analysis, we evaluated impedance telemetry data from 32 cochlear implant cases (16 with radiologically confirmed electrode array migration and 16 matched stable controls). A random forest regression model was trained to estimate the longitudinal position of the most basal electrode based on transimpedance matrices, cochlear dimensions, and implant parameters. Model predictions were compared against radiological ground truth, and migration was detected using a defined threshold of 1 mm. Electrode array migration was detected with a sensitivity of 88% and a specificity of 100%. Migration was identified in clinically inconspicuous cases with largely unremarkable impedance trajectories and across long-term follow-up. In addition to electrode emigration, postoperative imaging revealed non-surgically induced electrode immigration, a phenomenon not previously reported. Impedance-based machine learning could enable radiation-free detection of cochlear implant electrode array migration during routine follow-up. This low-burden approach has the potential to support earlier clinical review and intervention, particularly in pediatric patients. While this study represents a first proof-of-concept, validation on larger cohorts could allow this low-burden approach to be readily integrated into standard post-implantation care. The approach could enable earlier intervention and reduce revision surgeries in pediatric CI patients.

**Supplementary Information:**

The online version contains supplementary material available at https://doi.org/10.1007/s10916-026-02468-0.

## Introduction

Cochlear implants (CIs) are neuroprostheses that have revolutionized the treatment of severe to profound sensorineural hearing loss by directly stimulating the auditory nerve. With an incidence of 3 to 7%, migration of the implanted electrode arrays is a rare but recognized postoperative occurrence after (CI) surgery [1–4]. However, incidence is likely higher than reported, as array migration may remain undetected during CI follow-up, particularly when symptoms are mild and comparative radiological imaging is not performed systematically.

Migration of the CI electrode array can have multiple causes, including external forces (e.g., head trauma [5]), insufficient surgical fixation of the lead [3, 6], skull growth in children [7–9], and retraction forces associated with neo-ossification [10] or adhesions and fibrotic bands [11]. Subtle array migration can alter the effective CI frequency allocation and compromise speech understanding [3, 12], with displacements as small as 1 mm producing perceptible changes for patients [13]. In the worst case, migration requires revision surgery, which is estimated to account for approximately 1 to 15% of all revision procedures [14].

To mitigate the clinical impact of electrode array migration, continuous monitoring and timely intervention are essential. This is especially critical in pediatric patients, who not only face a higher prevalence of migration [15, 16] but also undergo a crucial period of speech and language development during early childhood [17]. Current detection methods, however, are reactive and non-scalable. Clinically, array migration is typically suspected only after nonspecific symptoms arise, such as loss of neural response telemetry, reduced speech understanding, device rejection, or unpleasant stimulation effects [3, 12, 14, 18]. While comparative imaging provides the most direct verification of electrode position, its use is limited by radiation exposure, high costs, and resource constraints, making it impractical for routine monitoring. Consequently, there remains a critical gap in objective, automated tools capable of enabling continuous, low-burden screening to detect migration early and facilitate prompt clinical review.

In this context, impedance telemetry, routinely acquired during CI follow-up or remotely performed by the patient [19, 20], could provide a practical approach for continuous screening. It is rapid, cost-efficient, and avoids exposure to ionizing radiation, a consideration particularly relevant for pediatric patients [21]. Electrode impedances change over time due to foreign body reaction, inflammatory processes, and the formation of intracochlear tissue [22–26]. Based on prior evidence linking array insertion depth to impedance-related characteristics [27], we hypothesized that postoperative electrode array migration produces detectable changes in impedance [2, 14].

Machine learning and artificial intelligence are increasingly integrated into clinical workflows, with recognized potential in medical diagnostics [28]. In otolaryngology, however, adoption of data-driven machine and deep learning models remains limited [29, 30]. A recent case report of pronounced electrode array migration in a pediatric patient demonstrated that an impedance-based machine learning model could have prompted revision surgery approximately 1.5 years earlier [18, 27]. Motivated by these findings, we evaluated the impedance-based approach in a larger, multi-center cohort. The aim of this study is to assess the clinical utility of an impedance-based model for detecting electrode array migration during post-implantation follow-up.

## Methods

### Study Design and Demographics

We retrospectively analyzed cochlear implantation cases from four CI centers, including cases with radiologically confirmed electrode array migration and matched controls, implanted between 2011 and 2025. The analysis was approved by the institutional review board (ID 2023-367-S-SR). Data from patients implanted with lateral wall electrode arrays (FLEX series; MED-EL GmbH, Innsbruck, Austria) were included in the analysis. In addition, each patient was required to have at least two radiological scans (i.e., X-ray projection or three-dimensional computed tomography (CT) or cone beam CT scans) of the same ear, obtained at different time points after cochlear implantation. We excluded cases with abnormal conditions of the cochlea (otosclerosis, enlarged vestibular aqueduct, or Ménière’s disease). In addition, we excluded single impedance recordings from the analysis with one or more contacts with a short circuit or open circuit as detected by the manufacturer’s telemetry software (MAESTRO, MED-EL GmbH). In all cases, cochlear implantation was performed using a conventional or extended round window (RW) approach [31]. Each case with radiologically confirmed array migrationwas matched to a stable control based on electrode array type, age at surgery, sex, and implanted ear side. Surgical techniques for electrodearray fixation included application of soft tissue (fat or fascia) and mechanical fixation using titanium clips [32].

### Computed Tomography Data

The first and second postoperative radiological scan were compared to assess differences in electrode array position. Three-dimensional and X-ray projections were available in 20 and 12 cases, respectively. In case of CT or cone beam CT scans, we used the open-source software 3D Slicer [33] to measure cochlear dimensions and to determine electrode contact positions relative to the round window membrane. In case of X-ray projections, the positions of cochlear electrode were determined using the available DICOM viewer (Sectra IDS7 v25.2, Sectra AB, Linköping, Sweden).

### Impedance Telemetry Data

For each case, we retrieved all impedance telemetry recordings available. All impedance data were recorded using the standard clinical telemetry protocol of the manufacturer’s telemetry software (MAESTRO, MED-EL, Austria) with an amplitude and pulse width of approximately 302 *μ *A and 24 *μ *s, respectively. The voltage at the recording electrode was measured in monopolar mode with respect to the ground electrode on the implant housing. Measurements were taken at the end of the second phase of the charge-balanced biphasic pulse [34]. We obtained (trans-)impedances (in k*Ω *) by dividing the recorded voltages (in mV) by the stimulation current (in *μ *A). For days with more than one impedance recording, only the latest recording was included discarding earlier measurements affected by short circuit or open circuit electrode faults.

### Impedance-based Electrode Position Estimation

All cases of the training set were required to have a preoperative and postoperative CT scan. In contrast to the test set from four CI centers, the cases of the training set for model evaluations originated from a single center due to availability of high resolution CT scans. We followed the impedance-based feature extraction and target definition described in [27] to estimate the position of the most basal electrode of the CI. Briefly, a machine-learning model predicts the insertion depth of the most basal electrode in millimeters from features extracted from the intraoperative voltage matrix and cochlear dimensions measured from preoperative CT images. The linear insertion depth for model training was extracted as follows: we extracted ground truth electrode insertion depth from CT scans using the open source, multiplanar reconstruction software 3D Slicer [33]. The electrode insertion depth was determined from postoperative CT scans. To minimize errors caused by misalignment of the image data, we co-registered the postoperative CT scan to the corresponding preoperative CT scan. Electrode coordinates were transformed into a local coordinate system [35], using a z-axis identified through robust detection of the cochlear modiolar axis [36, 37] from the preoperative CT scan. Manual labeling of electrode contacts and the calculation of linear insertion depth (in millimeters) from the round window along the CI’s internal lead were performed according to the method outlined in [38]. We extracted features from the full transimpedance matrix, derived cochlear-dimension features from preoperative CT scans (i.e., cochlear base length, width, and height in mm), and included CI parameters (i.e., stimulation current in *μ *A and ground path impedance in *Ω *), following [27]. The resulting features and target variable were used for subsequent model evaluations.

**Fig. 1:**
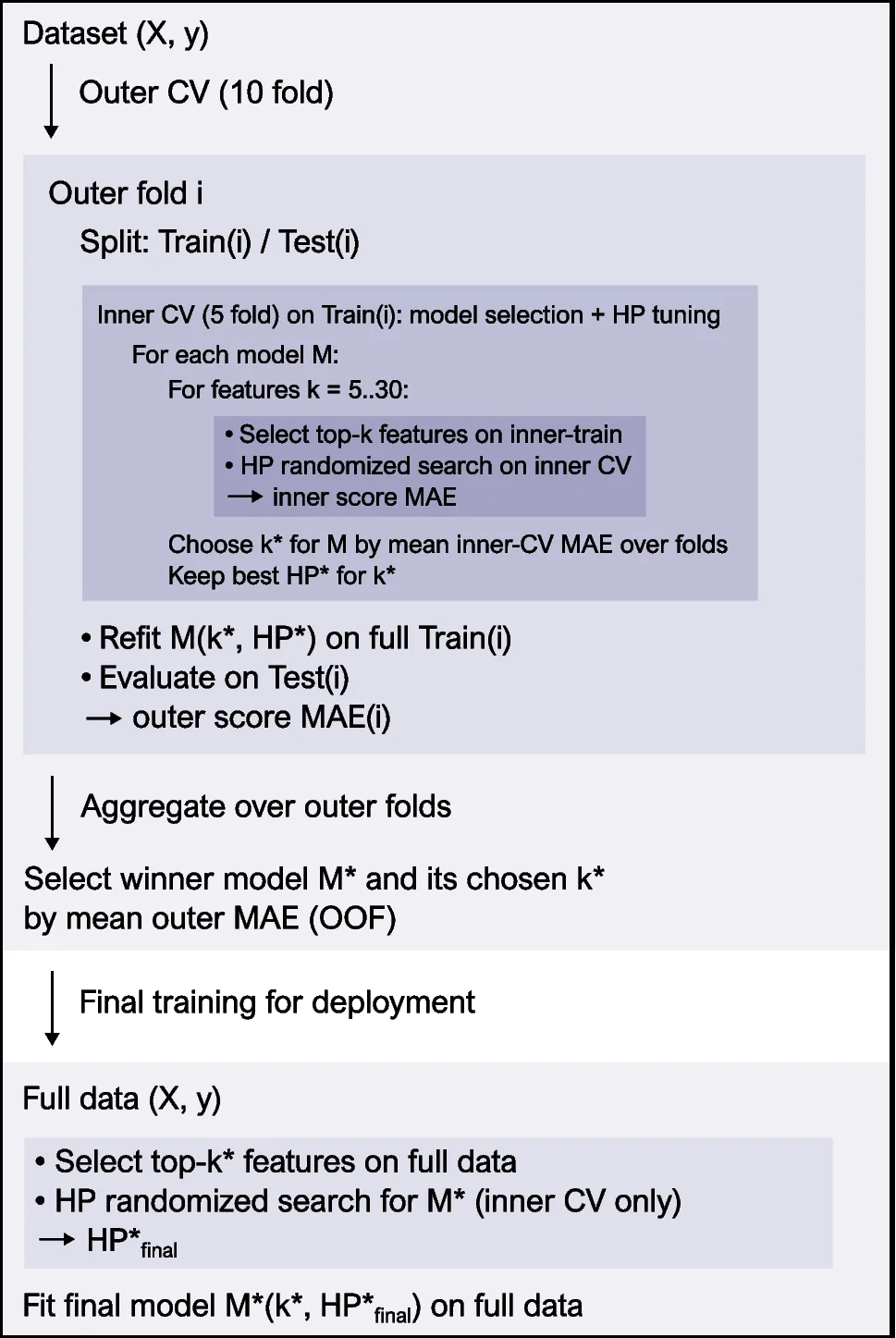
Pipeline for selecting the optimal model configuration for electrode position estimation. Model class, number of features, and hyperparameters were evaluated using nested cross-validation. The best configuration was then used to train a final model on the full dataset. CV, cross-validation; OOF, out-of-fold predictions; MAE, mean absolute error

Because the original model was trained on a single electrode array type (FLEX$^{28}$, MED-EL), we optimized the approach for model evaluation and retrained the model on an expanded cohort of 179 cases including additional electrode array types from the same manufacturer (FLEX portfolio and Standard arrays). The model evaluation pipeline is shown in Fig. 1. We used nested cross-validation with ten outer folds and five inner folds for unbiased performance estimation and model selection. Six regression models were evaluated: linear regression, lasso regression, support vector regression, extreme gradient boosting, extremely randomized trees (extra trees), and random forests. In each outer fold, the data were split into a training set and a held-out test set. Feature-wise standardization (z-scoring) was fitted exclusively on the outer-fold training set and applied to both training and test data. Within the inner cross-validation loop, conducted on the outer-fold training data, we jointly optimized the number of selected features *k* and the model hyperparameters. For each candidate *k**∈ * {5,...,30} (step size 1), feature selection was performed within each inner-fold training split by selecting the top-k features based on F-statistic score. Hyperparameters were tuned using randomized search, and the optimal configuration was selected based on inner-fold performance. For each model class, the value of *k* features was selected based on the aggregated inner-fold performance (mean across folds). The model was then refit on the full outer-fold training set using the selected *k* features and the tuned hyperparameters, and evaluated on the outer-fold test set. The final model class was chosen as the one with the lowest mean absolute out-of-fold error. Finally, we performed a randomized hyperparameter search on the full dataset using the selected model class and its chosen *k* features to obtain the hyperparameters of the final model. To prevent data leakage, we retrained separate prediction models for each of the 32 study cases using the best-performing configuration, excluding the respective case from the training. This was necessary because some cases in the training dataset overlapped with the study cohort.

### Definition and Detection of Electrode Array Migration

The impedance-based position estimation model introduced in [27] calculates the position of the most basal electrode. Therefore, ground truth array migration was defined as an absolute longitudinal displacement of the most basal electrode exceeding 1.0 mm (migration detection threshold). This threshold was chosen to exceed the expected measurement noise of the radiological reference and to minimize false detections due to imaging resolution and slice spacing. In our training set of the position estimation model, CT scans had an average in-plane resolution of 0.2 mm (maximum 0.5 mm) and an average slice spacing of 0.27 mm (maximum 1.0 mm). Electrode displacements below this threshold are more likely attributable to imaging-related uncertainty than true electrode array migration. The most frequent FLEX$^{28}$ electrode array in our dataset has an active stimulation range of 23.1 mm between the first and last of the 12 electrodes. The chosen migration detection threshold is therefore on the order of half an inter-electrode spacing and comparable with that of [39]. When the displacement was smaller, the electrode array was considered to be in a stable position. Transverse electrode displacement (e.g., towards the modiolar or lateral wall) was not assessed as part of this study.

We used the case-specific models to perform impedance-based detection of electrode array migration. For each impedance recording, the model takes the transimpedance matrix, cochlear dimensions, and CI parameters as input and outputs an estimate of the position of the most basal electrode (in mm), yielding one position estimate per recording. Migration was assessed by comparing each estimate to a baseline estimate: initially, the baseline was the position estimate based on the first (i.e., intraoperative) impedance recording. For each subsequent recording, we computed the signed change in estimated basal-electrode position relative to the current baseline and flagged migration when this change exceeded the defined detection threshold of 1 mm. We distinguished between emigration (electrode movement out of the cochlea; negative change) and immigration (electrode movement further into the cochlea; positive change). After a migration event was detected, the corresponding position estimate was used as the new baseline for evaluating later recordings.

The operation of the proposed position estimation model is illustrated using the position estimates in Fig. 5. For visualization, the first estimate of each case was offset to 0 mm to enable consistent cross-case comparison. The model detects an electrode array migration event when a position estimate falls outside the electrode detection margin (shaded in light blue). Events are classified as emigration (downward arrows on orange background; e.g., S03 and S16) or immigration (upward arrows on green background; e.g., S13) depending on the direction of displacement. If all position estimates for a case remain within the detection margin, the array is classified as stable (e.g., S10).

Finally, we compared model-based detections with the ground-truth occurrence of electrode array migration and evaluated performance using sensitivity (true-positive rate), specificity (true-negative rate), and receiver operating characteristic area under the curve (ROC AUC). For performance evaluation, we ignored migration direction and treated migration as a binary outcome per case (unsigned migration present/stable). This decision was driven by the imbalance of the training dataset, which was dominated by emigration cases. Direction-specific evaluation of immigration cases would have been based on insufficient observations to yield reliable performance estimates. Consequently, the reported performance metrics reflect combined migration detection accuracy regardless of direction. This is clinically justified since both electrode emigration and immigration have clinically relevant consequences. Moreover, we did not assess temporal accuracy. Detections were not required to fall between the two postoperative CT scans that bound the interval in which migration must have occurred. Marginal deviations from this interval may partly reflect measurement asynchrony rather than true temporal inconsistency. This arises from the fact that impedance data were recorded at regular clinical intervals while CT scans were performed on clinical indication only, and were therefore not always acquired on the same day. We evaluated performance at the case-level such that, for radiologically confirmed migration, the detection of at least one migration event was sufficient for a true-positive classification, irrespective of the number of detections.

### Influence of Electrode Array Migration on Impedances

To assess the effect of electrode array migration on clinical impedances (i.e., the diagonal entries of the transimpedance matrix), we compared group-level impedances across all 12 electrode contacts. For each electrode, impedances in control and migration cases were contrasted at two time points: (i) the intraoperative (surgical) impedance recording and (ii) the impedance recording corresponding to the first migration event detected by the position estimation model. Since electrode array migration occurred between successive postoperative radiological scans in all migration cases, the first model-detected event was selected over the second postoperative scan to more accurately reflect the impedance profile at the actual time of migration. For control cases, the postoperative impedance recording closest in time to the corresponding migration event was used for comparison.

We tested for differences in central tendency of impedance values between the two groups (control vs. migration) using a two-sided Mann–Whitney U test on independent samples. The test was performed separately for the intraoperative recording and for the first model-detected migration event. The significance level was set to *α *=0.05. In addition, we computed 95% confidence intervals for pairwise median differences [40].

## Results

### Radiologically Confirmed Electrode Array Migration

Cases of array migration or stable electrode positions were confirmed by comparing two postoperative radiological images. Applying the inclusion and exclusion criteria, 16 migration cases and 29 stable cases were identified. The requirement for two postoperative CT scans was the most restrictive criterion and accounted for the majority of exclusions. For analysis, each migration case was matched to one stable control case, yielding 32 cases (16 migration, 16 controls). These cases corresponded to 28 patients, including four bilateral implantations (13 females and 15 males; Table 1). Patients’ age ranged from 1 to 89 years (median age 56 years) and there were 15 left and 17 right-sided implantations.

**Table 1:** Demographic details of the study cohort. F, female; M, male; R, right; L, left; Superscript c: control, Age, Age at surgery in years

ID	Side	Age	Sex	Array	ID	Side	Age (yr)	Sex	Array
S01	R	38	F	FLEX$^\text {soft}$	S09	R	44	F	FLEX$^{28}$
S01$^{c}$	L	77	M	FLEX$^\text {soft}$	S09$^{c}$	R	82	M	FLEX$^{28}$
S02	L	69	M	FLEX$^\text {soft}$	S10	R	61	F	FLEX$^{28}$
S02$^{c}$	R	68	F	FLEX$^\text {soft}$	S10$^{c}$	L	65	F	FLEX$^{28}$
S03	R	28	M	FLEX$^\text {soft}$	S11	L	55	M	FLEX$^{28}$
S03$^{c}$	L	40	F	FLEX$^{28}$	S11$^{c}$	R	57	M	FLEX$^{28}$
S04	L	53	F	FLEX$^\text {soft}$	S12	R	48	M	FLEX$^{28}$
S04$^{c}$	L	45	M	FLEX$^{28}$	S12$^{c}$	R	47	F	FLEX$^{28}$
S05	R	54	F	FLEX$^{28}$	S13	R	89	M	FLEX$^{28}$
S05$^{c}$	R	60	F	FLEX$^{28}$	S13$^{c}$	L	46	F	FLEX$^{28}$
S06	R	63	M	FLEX$^{24}$	S14	L	1	F	FLEX$^{28}$
S06$^{c}$	R	76	M	FLEX$^{26}$	S14$^{c}$	R	1	F	FLEX$^{28}$
S07	R	51	M	FLEX$^{24}$	S15	R	39	M	FLEX$^\text {soft}$
S07$^{c}$	L	69	M	FLEX$^{28}$	S15$^{c}$	L	75	M	FLEX$^{34}$
S08	L	53	M	FLEX$^{28}$	S16	L	72	F	FLEX$^{28}$
S08$^{c}$	L	71	F	FLEX$^{28}$	S16$^{c}$	L	73	F	FLEX$^{28}$

**Fig. 2:**
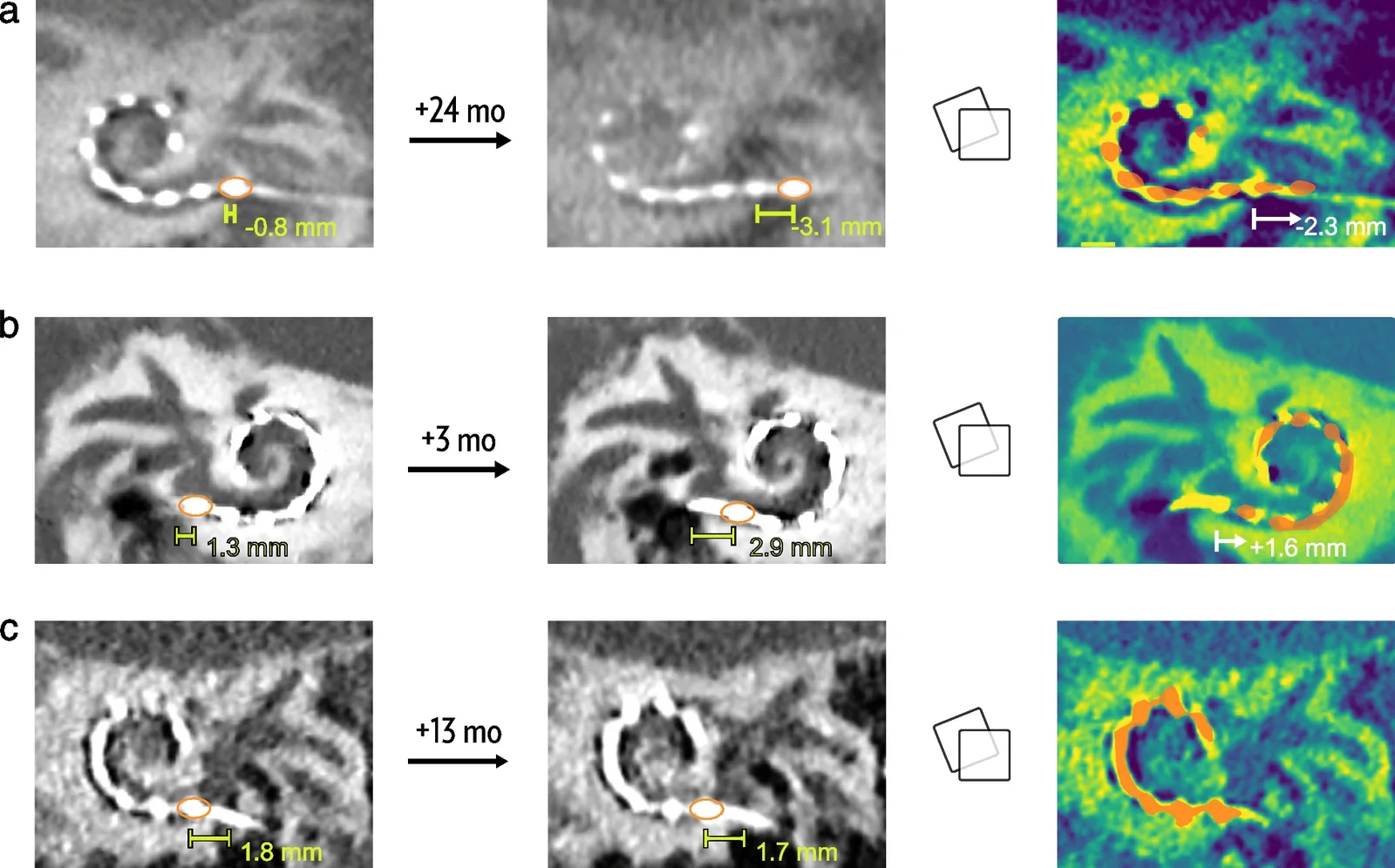
Comparison of two postoperative computed tomography (CT) scans after cochlear implantation. (**a**) Case with emigration of the electrode array (S11). (**b**) Case of electrode immigration into the cochlea (S13). (**c**) Case with stable electrode array position (S03$^c$). Distances indicate the linear insertion depth (in mm) of the most basal electrode (circled in orange) with respect to the round window. The time interval between the first and second postoperative CT scan is given in months (mo). The third column shows an overlay of the two postoperative CT scans, indicating the electrode positions in the first and second scan in yellow and orange, respectively. The distance measure indicates the difference in linear insertion depth between the most basal electrodes in both scans

Figure 2 shows representative cases of array emigration, immigration, and stable electrode positions. In the emigration case (S11; Fig. 2a), the most basal electrode (E12) moved by -2.3mm, resulting in two extracochlear electrodes. This corresponds to a change in angular insertion depth of the most apical electrode (E1) by -86$^{\circ }$. The event of electrode emigration occurred within a time interval of 24 months. In the immigration case (S13; Fig. 2b), the electrode array moved deeper into the cochlea, with a linear displacement of the most basal electrode (E12) of +1.6 mm (angular displacement of the most apical electrode E1 of +50$^{\circ }$) and the electrode immigration occurring within a time interval of 3 months. In control case (S03$^c$ Fig. 2c), the electrode array remained in a stable position over a time interval of 13 months.

### Influence of Electrode Array Migration on Impedances

**Fig. 3:**
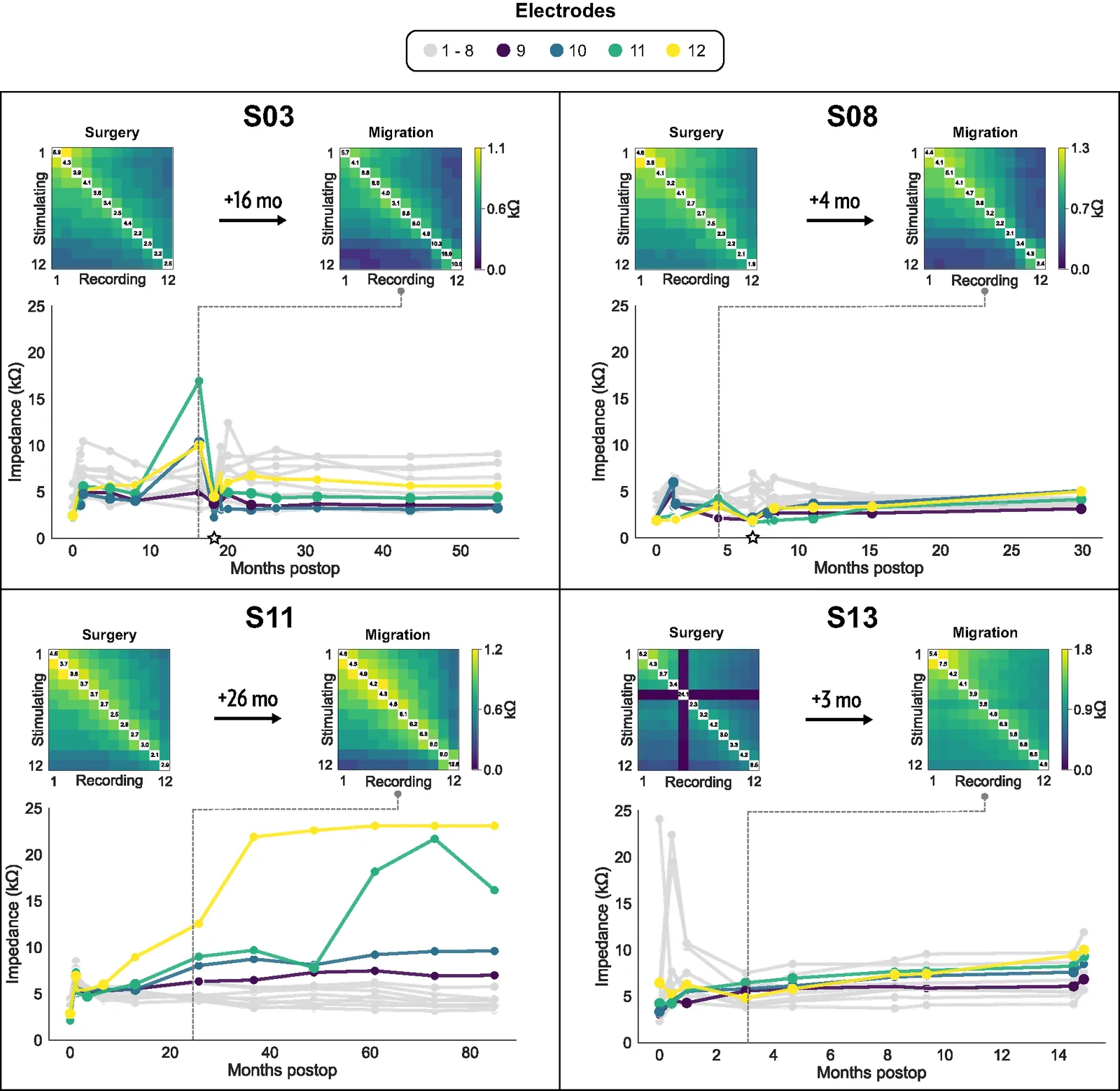
Progression of clinical impedances and transimpedance matrices over time in cases with radiologically confirmed electrode emigration (S03, S08, S11) and immigration (S13). Each panel displays, at the top, the transimpedance matrices recorded during surgery and at the time of radiologically confirmed migration, with the interval between scans indicated in months (mo). On the bottom, clinical impedances (k*Ω *) are shown, with the four most basal electrodes color-coded (E1-E12, apical-basal). The time point of confirmed migration is indicated by a vertical gray dashed line, and a white star on the x-axis marks a performed revision surgery (cases S03 and S08)

Figure 3 illustrates the progression of clinical impedances over time and compares transimpedance matrices recorded during surgery with those recorded at the time of the radiologically confirmed array migration. The impedance trajectories in the figure show representative patterns observed in multiple cases with confirmed electrode array migration, including sharp rises, fluctuations, gradual increases, and largely stable trajectories with minimal variation. Clinical impedances of the emigration case S03 exhibited a sharp rise in impedance at the second most basal electrode (E11) at the time of confirmed migration. In the emigration case S08, impedances fluctuated but showed only limited overall change around the time of confirmed migration and revision surgery. Consistent with the impedance trajectories, both cases S03 and S08 demonstrated increased values in the immediate off-diagonal entries of the transimpedance matrix at the time of confirmed migration relative to the intraoperative recording. Moreover, the diagonal-to-off-diagonal gradient became much steeper, especially in the area of stimulating electrodes E10-E12 and recording electrodes E1-E9.

In the immigration case S13, an open circuit was present at the medial electrode (E5) in the intraoperative recording and persisted until the wound-control visit two weeks after cochlear implantation. Following CI activation one month postoperatively, clinical impedances returned to normal levels. Finally, in the emigration case S11, the two most basal electrodes (E11-E12) exhibited a strong, gradual impedance increase over time.

The maximum follow-up time of impedance telemetry recordings after cochlear implantation ranged from 3 to 146 months, with a mean of 34 months. We excluded 42 of 395 impedance recordings (11%) from the analysis due to bad contact status (i.e., one or more electrode contacts with an open or short-circuit). The majority of these (24) were from a single patient, S16, with the longest follow-up time (Fig. 5). In S03$^c$, S13, and S16, bad contact status led to the exclusion of the intraoperative impedance recording.

**Fig. 4:**
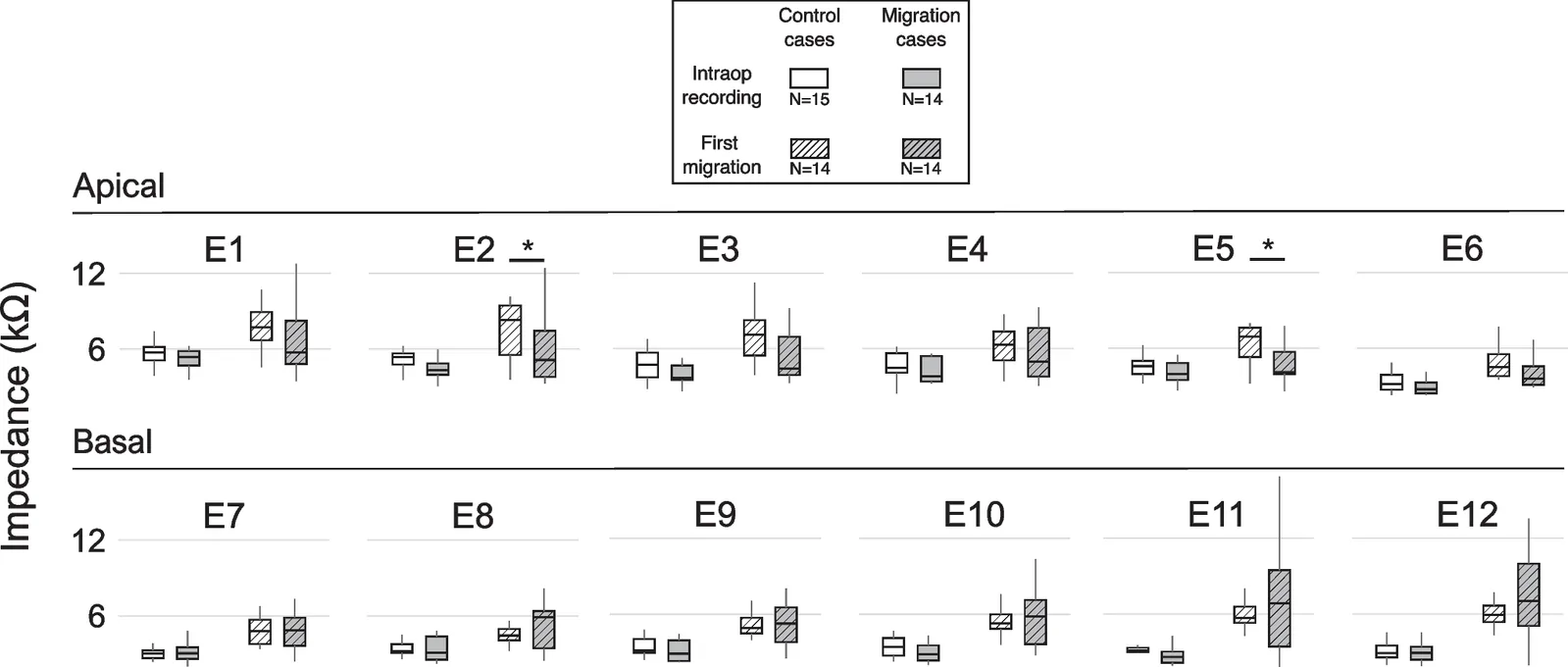
Boxplot comparison of clinical impedances (in k*Ω *) between control (stable) and migration (dislocated array) cases, grouped by apical (E1-E6) and basal electrodes (E7-E12). Impedances are shown for each electrode at intraoperative recording (open boxes) and at the first model-detected migration event (hatched boxes). Impedance recordings of control cases were time-matched to the nearest postoperative measurement. Sample sizes are reported in the legend; *p <.05

We analyzed clinical impedances on a group level to assess differences in control cases compared to migration cases at two different time points (Fig. 4). As for the position estimation model, single samples were excluded for the intraoperative impedance recordings due to bad contact status (control cases: -1, migration cases: -2). In addition, two samples were missing and excluded in both groups for the impedance recording at the first model-detected migration event due to false negatives (sample sizes in the legend of Fig. 4; false negatives in supplemental Fig. S1).

Clinical impedances were comparable between control and migration cases at the intraoperative recording (Fig. 4). At the first model-detected migration event, control cases exhibited slightly higher clinical impedances at the apical electrodes (e.g., E1–E3) than migration cases, whereas the trend reversed for the most basal electrodes (e.g., E10-E12), where migration cases showed slightly higher impedances than controls. For these basal electrodes, migration cases also displayed greater impedance variability. Across all 12 electrodes and time points, two comparisons showed statistically significant group differences in impedance distributions between control and migration cases. The strongest effect was observed for the medial electrode E5 at the first model-detected migration event, with median impedances of 4.1 k*Ω * vs. 6.9 k*Ω * in migration and control cases, respectively (2.8 k*Ω * median difference; 95% confidence interval: 0.3 to 3.3 k*Ω *; Mann-Whitney *U=152*, *p=0.014*; Fig. 4). The second-strongest difference was observed for the apical electrode E2 at the first migration time point (3.2 k*Ω * median difference; 95% confidence interval: 0.1 to 4.4 k*Ω *; *U=143*, *p=0.041*).

### Estimation Model

The model evaluation identified a random forest regressor with *k*=15 features as the best performing model. Compared to the ground truth from CT scans, the best performing model predicted the position of the most basal electrode with a performance of 0.94 mm ± 0.75 mm (mean ± standard deviation of out-of-fold mean absolute error (MAE)) and an error range of −3.5 to 2.4mm. The worst model was support vector regression with 30 features and a performance of 1.0 mm ± 0.78 mm. Impurity-based feature importances (i.e., how much, on average, each feature improves splits in the forest) identified i) the impedance gradient phase at recording electrode 8 with stimulation electrode 11, ii) the impedance decay rate in basal direction with stimulation electrode 8, and iii) the impedance magnitude at recording electrode 10 with stimulation electrode 12 as the three most important features. The optimal hyperparameters fitted on the full training set for the best random forest model and the parameter search space for all models evaluated are shown in supplemental Table S1.

### Automatic Detection of Electrode Array Migration

On average, the first migration event was detected 10.6 months after initial surgery in 14 cases (min: 0.2 months, max: 41.7 months). For migration cases, the estimated linear insertion depth of the most basal electrode with respect to the baseline of 0 mm in the first recording ranged from −3.7 to 3.0 mm (mean of −0.1 mm). For control cases, the estimated positions ranged from −0.5 to 0.7 mm (mean of 0.1 mm).

In total, we estimated the electrode positions over time in 32 cases (16 migration and 16 control cases) using the impedance-based model (Fig. S1 in the supplemental material). The comparison of ground truth array migration status with model predictions is shown in Table 2. The model correctly detected an array migration in 14 out of 16 cases with radiologically confirmed migration. In the remaining two cases, the model did not detect the presence of electrode array migration (false negative). All stable control cases were estimated as such. There were no false positives. Overall, the model reached a ROC AUC of 94%, a sensitivity of 88%, and a specificity of 100% (14 true positives, 0 false positives, 16 true negatives, 2 false negatives).

**Table 2:** Ground truth vs. model predictions for migration and control cases. Numbers in parentheses show migrated electrodes. Stars (*) indicate cochlear revision surgery due to array migration. S01 had two revisions for repeated dislocation. TP, true positive; FN, false negative; TTM, time to migration (months); ROC AUC, receiver operating characteristic area under the curve

Migration cases					Control cases
ID	Ground Truth	TTM	Prediction	Outcome	Prediction
S01**	Emigration (2)	20	Emigration	TP	Stable
S02	Emigration (2)	23	Emigration	TP	Stable
S03*	Emigration (2)	16	Emigration	TP	Stable
S04*	Emigration (2)	13	Immigration	TP	Stable
S05*	Emigration (2)	18	Emigration	TP	Stable
S06*	Emigration (3)	14	Emigration	TP	Stable
S07*	Emigration (2)	13	Immigration	TP	Stable
S08*	Emigration (2)	4	Emigration	TP	Stable
S09	Emigration (1)	46	Stable	FN	Stable
S10	Emigration (1)	41	Stable	FN	Stable
S11	Emigration (1)	25	(E/Im-)migration	TP	Stable
S12	Immigration (1)	3	(E/Im-)migration	TP	Stable
S13	Immigration (1)	3	Immigration	TP	Stable
S14	Emigration (4)	22	Emigration	TP	Stable
S15	Emigration (1)	42	Emigration	TP	Stable
S16	Emigration (4)	146	Emigration	TP	Stable
Overall performance: TP: 14, FP: 0, TN: 16, FN: 2, ROC AUC: 94%, Sensitivity: 88%, Specificity: 100%					

**Fig. 5:**
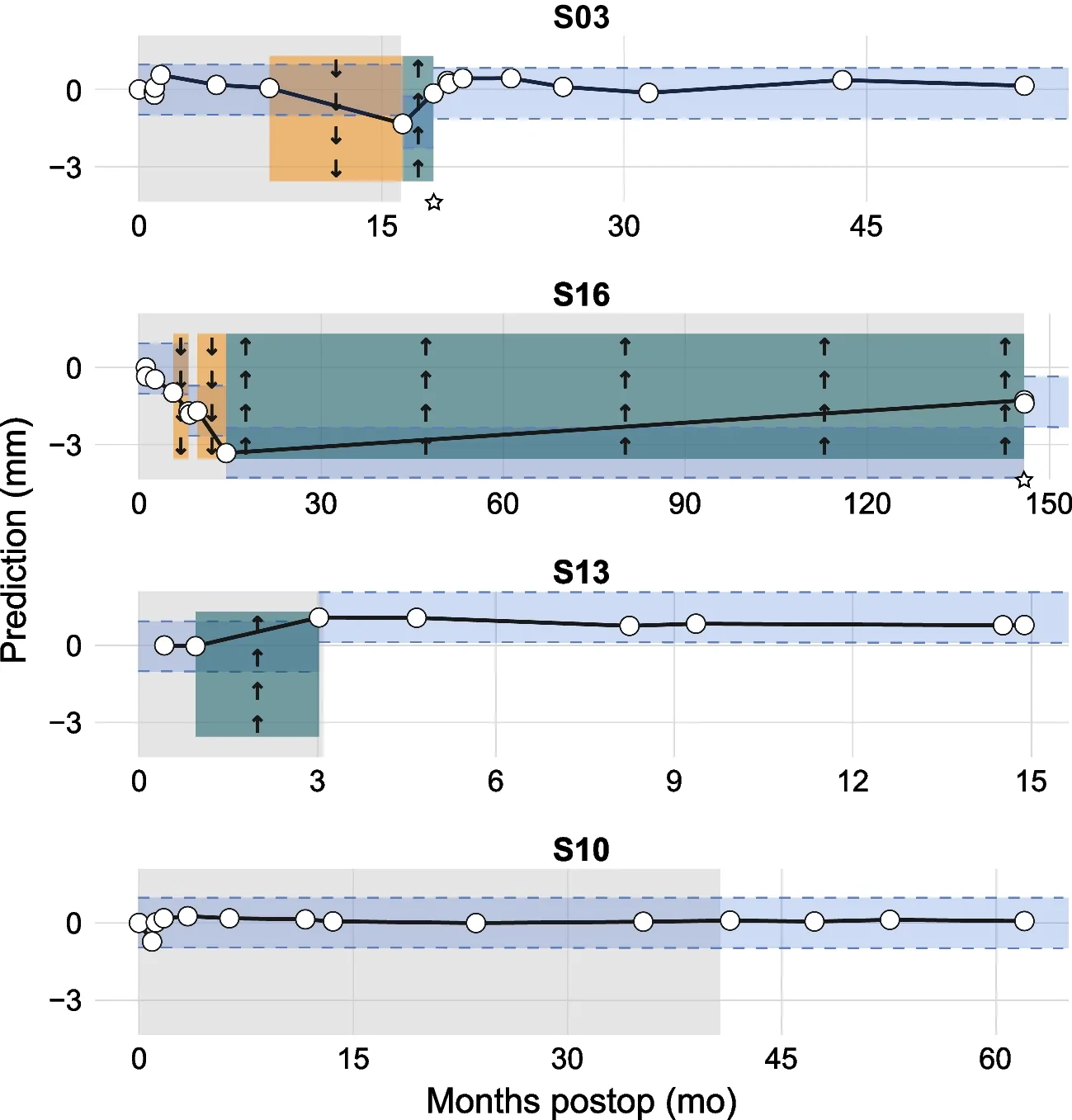
Estimated positions (in mm) of the most basal electrode over time for four out of 32 cases. The first estimate is offset to 0 mm to enable consistent cross-case comparison. Detected emigration and immigration events are indicated by downward arrows on an orange background and upward arrows on a green background, respectively. For clarity, we highlight only the interval between the last impedance recording within the detection margin and the first subsequent estimate outside the margin (rather than the baseline estimate itself). The migration detection margin is shown in light blue (± the migration detection threshold of 1 mm). For S13, the intraoperative position estimate is missing due to an open circuit in one electrode. The gray shaded area denotes the time to migration in months, defined as the interval between initial surgery and radiologically confirmed array migration. The time point of a revision surgery is indicated by a white star on the x-axis (cases S03 and S16)

Figure 5 shows the model estimates for four example cases with electrode emigration (S03 and S16), electrode immigration (S13), and stable electrode array position (false negative for S10). The additional events of electrode immigration for case S03 (month 18) and S16 (month 146) were induced by the revision cochlear surgery to fully re-introduce the electrode array again into the scala tympani.

## Discussion

### Detection of Electrode Array Migration

In the cochlea, electrode displacements of ${2\,\mathrm{\text {m}\text {m}}}$ can cause a shift of approximately half an octave in the apical region (e.g., ${300\,\mathrm{\text {Hz}}}$ to ${450\,\mathrm{\text {Hz}}}$) or a quarter octave in the basal region (e.g., ${4\,\mathrm{\text {k}\text {Hz}}}$ to ${5\,\mathrm{\text {k}\text {Hz}}}$) [41, 42]. Even small electrode displacements of ${1\,\mathrm{\text {m}\text {m}}}$ can be negatively perceived by patients [13]. To mitigate potential adverse effects on speech perception, language development, and to facilitate timely acceptance of the CI audio processor, early detection and intervention are essential.

The position estimation model enables continuous, remote monitoring of electrode array migration, including cases where interpretation of standard clinical impedance measurements show no notable changes. Compatible with cloud-based or device-integrated platforms, the framework allows real-time data acquisition and longitudinal tracking across large patient populations, reducing the need for frequent in-clinic visits. Manufacturers already provide tools for remote impedance measurement, facilitating seamless integration of the model into routine post-implantation follow-up. This approach supports early, radiation-free screening and is well suited for telehealth, as impedance telemetry can be acquired by patients at home using existing cochlear implant hardware. Intended as a clinical decision support tool, the model’s primary purpose is to notify the clinical engineer and audiologist of possible array migration, suggesting that further clinical review may be warranted.

For example, in case S11, clinical impedances gradually increased after CI activation one month postoperatively (Fig. 3). Electrode migration was subsequently confirmed radiologically 26 months after surgery based on the second postoperative CT scan. The proposed model identified an emigration event as early as 13 months after surgery (Fig. S1) - 13 months before radiological confirmation - demonstrating the potential for earlier clinical review than would typically occur based on postoperative imaging or visual inspection of impedances alone. A clinician relying solely on visual inspection might not have suspected migration until month 26 (radiological confirmation) or even later, when the most basal electrode (E12) exceeded 20 k*Ω * at month 37. Notably, despite the elevated impedance at E12, the transimpedance matrix at the time of radiologically confirmed migration (month 26) did not display a clearly abnormal pattern (Fig. 3).

In addition, the position estimation model detected electrode array migration in several other clinically relevant scenarios: (i) cases with largely unremarkable impedance trajectories (e.g., case S08; Fig. 3), (ii) long-term follow-up spanning more than 12 years (e.g., case S16; Fig. 5), and (iii) spontaneous electrode array migration followed by surgically induced migration associated with revision surgery (e.g., the two consecutive migration events in case S03; Fig. 5).

The small difference in performance between the best and worst estimation models (a difference of 0.06 mm in mean MAE) suggests that the choice of machine learning algorithm had only a limited impact on prediction accuracy and that performance was instead primarily determined by the information content of the input features themselves. This is consistent with the notion that impedance-based features contain a finite amount of positional information, beyond which increasing model complexity offers little additional benefit.

### Occurrences of Electrode Immigration

Electrode emigration, defined as postoperative array migration out of the cochlea, is a well-recognized complication. To our knowledge, this study is the first to report electrode immigration, i.e., migration in the opposite direction with the array advancing further into the cochlea after surgery. Electrode immigration was confirmed radiologically in 2 of 16 migration cases (13%), with the most basal electrode displaced by +1.4 mm (case S12) and +1.6 mm (case S13; Fig. 2b).

Both electrode emigration and immigration may arise from insufficient fixation of the CI’s internal lead [3, 43] in combination with external mechanical forces. Electrode emigration, in particular, has been additionally associated with skull and mastoid growth in children [7–9] and with retraction forces related to neo-ossification [10]. Immigration may plausibly occur when the lead cable is coiled too tightly within the mastoid cavity, such that a release of stored bending energy advances the array further into the cochlea. The proposed model was able to infer migration direction in most cases, although direction confusions occurred (e.g., case S07; Fig. S1). Direction-specific performance could likely be improved by expanding the training set to include more immigration cases, as the present dataset was imbalanced and dominated by emigration.

In case of electrode immigration, audiological consequences may be less severe because the electrodes remain within the cochlea and typically do not require deactivation. Nevertheless, immigration can shift the effective stimulation sites relative to the intended frequency allocation, increasing frequency mismatch and potentially impairing speech understanding [44–46].

### Influence of Array Migration on Impedances

Electrical impedances recorded by CI are a functional marker of the surrounding electrode-tissue interface and nearby anatomical structures. Figure 4 shows that migration and control cases had comparable impedance distributions in intraoperative recordings, indicating similar initial conditions for the electrode-tissue interface in both groups. At the first model-detected migration event, a systematic spatial pattern emerged: Impedances tended to be lower at the apical electrodes and higher at the basal electrodes in migration cases compared to control cases. This observation is consistent with the composition of the model-detected migration cohort, which predominantly included emigration cases (12 emigration cases versus 2 immigration cases). Emigration is expected to result in a shallower electrode insertion depth, i.e., toward the basal regions of the cochlea. The observed reduction in impedances may reflect the larger conductive perilymphatic space associated with the wider scala tympani following basal migration of the apical contacts, as well as a potentially increased distance from cochlear walls. This is because impedance is also influenced by the electrode’s position within the cross-sectional geometry of the scala tympani: proximity to either the modiolar or the lateral wall reduces the surrounding perilymphatic volume and increases impedance, while a more central position has the opposite effect. Elevated basal impedances may arise when basal contacts are positioned nearer the RW or extend extracochlearly, reducing electrical coupling to the perilymphatic space. In this context, a reduction in local conductivity in combination with increased fibrous tissue growth around these basal contacts could plausibly increase the measured impedances. The two electrodes showing the strongest group differences at the first model-detected migration time point (E2 and E5) are consistent with these findings (Fig. 4). In addition to the shift in the central tendency, migration cases showed greater dispersion at the two most basal electrodes (E11-E12). This increased variability is caused by a subgroup of cases with pronounced impedance elevation at the basal contacts, as can be seen in the example of case S03 (Fig. 3), where a sudden rise in basal impedances could be observed at the time of migration.

In routine clinical follow-up after cochlear implantation, audiologists and clinical engineers may suspect electrode array migration based on visual inspection of longitudinal impedance measurements. Two patterns that might prompt suspicion and warrant further clinical review are (i) abrupt impedance increases and (ii) gradual but persistent impedance elevations over time, particularly at basal electrodes [43] (e.g., cases S03 and S11; Fig. 3). However, such patterns were not consistently observed in all migration cases. For instance, cases S08 and S13 exhibited largely unremarkable impedance trajectories, comparable to those seen during uncomplicated follow-up, despite radiologically confirmed migration (Fig. 3). The proposed impedance-based position estimation model could help flag these otherwise inconspicuous cases and facilitate earlier clinical review before migration manifests with negative consequences for the patient.

Finally, electrical impedances may not sufficiently capture positional changes of the electrode array in all cases [47]. In cases S09 and S10 (Fig. 5 and Fig. S1), impedance trajectories remained within typical ranges and the model did not detect a migration event. In addition, the model might have missed electrode array migration because both cases involved only moderate displacement of the electrode array, with linear displacements of the most basal electrode (E12) of less than one inter-electrode spacing and therefore too little effect on impedances (S09: −1.6 mm, S10: −1.3 mm, both FLEX$^{28}$ arrays with inter-electrode spacing of 2.1 mm; Table 1).

### Limitations and Outlook

The position estimation model was trained on features derived from impedance telemetry, with ground truth electrode positions from CT scans. Most impedance measurements of the training set were acquired intraoperatively, whereas the corresponding CT scans were obtained either on the same day or shortly thereafter. Importantly, electrode array migration may also occur in the immediate postoperative interval between the intraoperative impedance recording and the postoperative CT scan. For example, approximately 10% of cases presented with early migration within the first two weeks after surgery [48]. Consequently, if postoperative CT imaging was delayed beyond the day of surgery or shortly after, these migration events remained undetected. This would introduce a mismatch between impedance features and the assumed electrode position, ultimately reducing the accuracy and precision of the model. The temporal evolution of impedances following implantation introduces model uncertainty that potentially reduced estimation accuracy. This could be mitigated by accounting for impedance changes attributable to inflammatory and foreign body reactions [25, 26, 49–51], which are unrelated to electrode position and therefore constitute a confounding signal. However, most features of the proposed model were based on off-diagonal values of the impedance matrix, which are less affected by polarization effects and immune-response-related impedance changes [25, 34, 38, 52–54]. Features therefore remained more stable over time following cochlear implantation, which strengthens confidence in the learned relationships between impedance input features and output electrode positions. The model was trained on a heterogeneous dataset of flexible electrode arrays from the same manufacturer, but both the overall sample size (*n = 179* training cases) and the representation of array types were limited, with a single type dominating (*n = 162* FLEX$^{28}$ , MED-EL). Given these constraints, the model may not yet have learned sufficiently generalizable features across array types or clinical centers. Training the model on a larger multi-center cohort with a more balanced representation of electrode array types would likely improve generalizability, allow identification of potential center bias, and enhance clinical applicability.

In the future, the proposed position estimation model could facilitate large-scale retrospective screening for electrode array migration in existing CI databases and provide a less biased estimate of migration incidence. Prospective integration into routine clinical follow-up and telehealth applications would enable evaluation of clinician acceptance and assessment of the method’s clinical utility for CI patients. Further validation on larger, external datasets is essential, including comparisons with clinician performance [55, 56]. Subsequent studies could also examine associations between electrode array migration and audiological outcomes, including speech understanding in quiet and noise, which were not addressed in the present work. Performance and robustness could be further enhanced by integrating impedance telemetry with complementary measures such as electrically evoked compound action potentials. Finally, incorporating features derived from impedance subcomponents [34, 57] and anatomical parameters [37] may further enhance the performance of the position estimation model.

## Conclusion

We demonstrate the clinical applicability of an impedance-based model for detecting migration of CI electrodes. The model accurately distinguished migration from stable positions. In addition, postoperative imaging revealed cases of electrode immigration, a positional shift further into the cochlea not previously reported. By enabling early detection during routine post-implantation follow-up and telehealth applications, the approach may support clinical engineers and audiologists in timely review and intervention. From a preventive perspective, secure fixation of the CI internal lead during surgery may reduce the risk of postoperative migration. As a first proof-of-concept requiring validation on larger cohorts, this approach nonetheless appears feasible for implementation into standard care, as the model relies on fast, cost-effective, and radiation-free impedance telemetry acquired directly from the implant.

## Supplementary Information

Supplementary information is available for this article.

## Supplementary Information

Below is the link to the electronic supplementary material.

Supplementary file 1 (pdf 1670 KB)

## Data Availability

The data analyzed in this study are not publicly available; however, they may be obtained from the corresponding author upon reasonable request.
