# Supplementary material for: Automated, Radiation-Free Detection of Cochlear Implant Electrode Array Migration Using Routine Impedance Telemetry
Source: J Med Syst. 2026 Nov 4;50(1):158. doi: 10.1007/s10916-026-02468-0 (PMC13736414; doi:10.1007/s10916-026-02468-0)
Supplement: Supplementary file 1 [file 10916_2026_2468_MOESM1_ESM.pdf]

Supplementary Information: Electrode Array Migration Detection

Table S1: Hyperparameter search space for all evaluated models using randomized search. For the random forest, the table additionally reports the optimal hyperparameters (x^*) fitted on the full training set using the best model configuration. HP: hyperparameter, RF: random forest.

Model	HP	Search space	x^* (RF only)
Random Forest	n_estimators	{100, 200, 300}	300
	max_depth	{None, 10, 20}	10
	min_samples_split	{2, 5, 10}	10
	min_samples_leaf	{1, 2, 4}	2
	max_features	{None, sqrt, log2}	log2
Extra Trees	n_estimators	{100, 200, 300, 500}	–
	max_depth	{None, 10, 20}	–
	min_samples_split	{2, 5, 10}	–
	min_samples_leaf	{1, 2, 4}	–
	max_features	{None, sqrt, log2}	–
	bootstrap	{True, False}	–
Support Vector Regression	C	{0.1, 1, 10, 100}	–
	epsilon	{0.01, 0.1, 0.2, 0.3}	–
	kernel	{rbf, poly}	–
	degree	{2, 3, 4}	–
	gamma	{scale, auto}	–
Lasso Regression	alpha	{0.001, 0.01, 0.1, 1, 10}	–
XGBoost	n_estimators	{300, 600, 1000}	–
	learning_rate	{0.01, 0.03, 0.05, 0.1}	–
	max_depth	{3, 6, 10, 16}	–
	min_child_weight	{1, 5, 10, 20}	–
	subsample	{0.6, 0.8, 1.0}	–
	colsample_bytree	{0.6, 0.8, 1.0}	–
	gamma	{0.0, 0.1, 0.5}	–
	reg_alpha	{0.0, 0.1, 1.0}	–
	reg_lambda	{0.0, 0.1, 1.0}	–

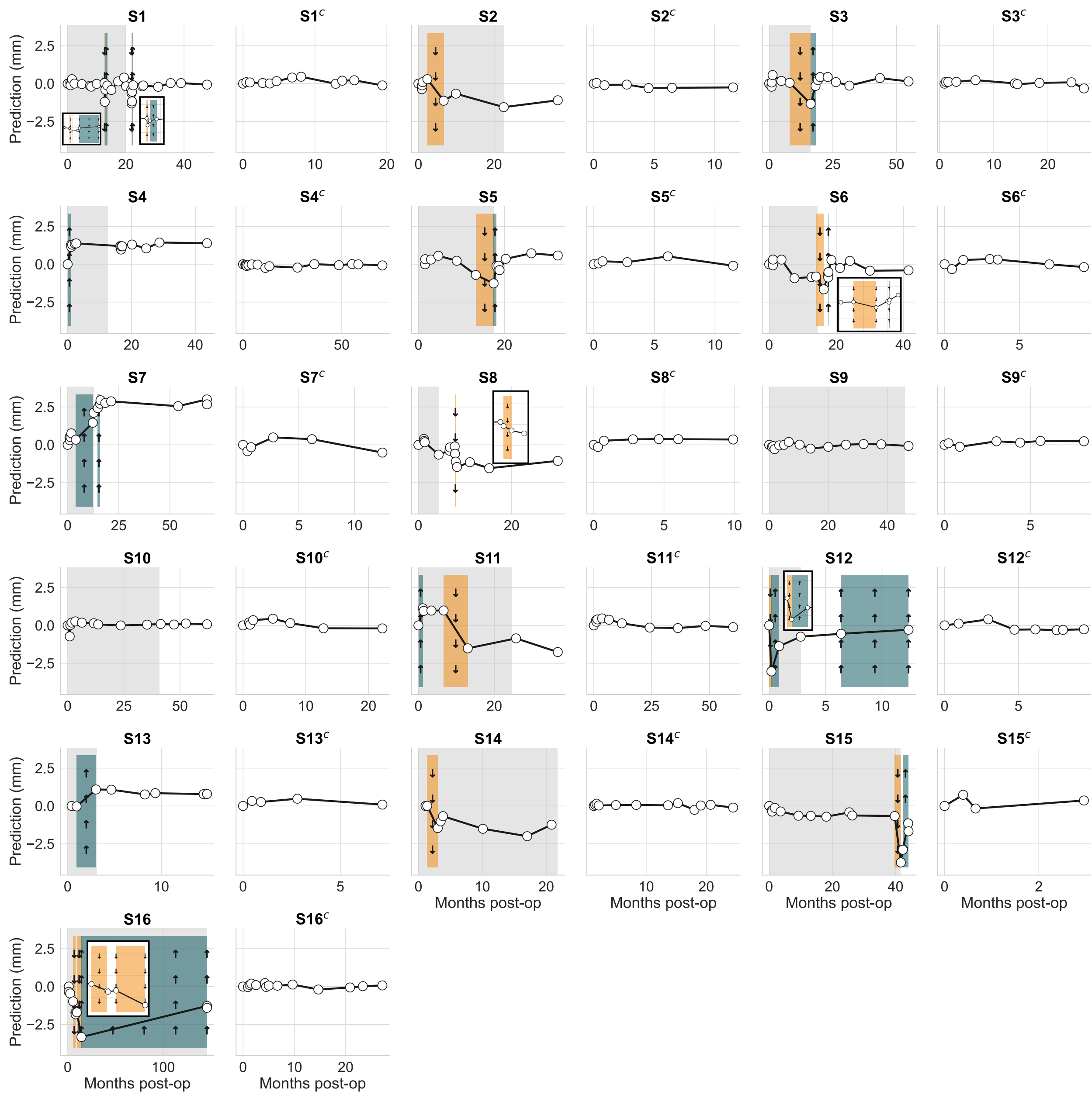

Figure S1: Estimated positions (mm) of the most basal electrode over time for all 32 cases (16 pairs of migration and control cases). Detected emigration and immigration events are indicated by downward arrows on an orange background and upward arrows on a green background, respectively. The gray shaded area denotes the time to migration in months, defined as the interval between initial surgery and radiologically confirmed electrode array migration. Superscript c: control.
